# Zinc-Chelating Compounds as Inhibitors of Human and Bacterial Zinc Metalloproteases

**DOI:** 10.3390/molecules27010056

**Published:** 2021-12-22

**Authors:** Fatema Rahman, Imin Wushur, Nabin Malla, Ove Alexander Høgmoen Åstrand, Pål Rongved, Jan-Olof Winberg, Ingebrigt Sylte

**Affiliations:** 1 Molecular Pharmacology and Toxicology, Department of Medical Biology, Faculty of Health Sciences, UiT—The Arctic University of Norway, NO-9037 Tromsø, Norway; fatema.rahman@uit.no (F.R.); imin.wushur@uit.no (I.W.); nabin.malla@uit.no (N.M.); jan.o.winberg@uit.no (J.-O.W.); 2 Department of Pharmaceutical Chemistry, School of Pharmacy, University of Oslo, NO-0316 Oslo, Norway; alexander.aastrand@gmail.com (O.A.H.Å.); pal.rongved@farmasi.uio.no (P.R.)

**Keywords:** bacterial virulence factors, matrix metalloproteases, zinc chelators, enzyme inhibition, docking, molecular interactions

## Abstract

Inhibition of bacterial virulence is believed to be a new treatment option for bacterial infections. In the present study, we tested dipicolylamine (DPA), tripicolylamine (TPA), tris pyridine ethylene diamine (TPED), pyridine and thiophene derivatives as putative inhibitors of the bacterial virulence factors thermolysin (TLN), pseudolysin (PLN) and aureolysin (ALN) and the human zinc metalloproteases, matrix metalloprotease-9 (MMP-9) and matrix metalloprotease-14 (MMP-14). These compounds have nitrogen or sulfur as putative donor atoms for zinc chelation. In general, the compounds showed stronger inhibition of MMP-14 and PLN than of the other enzymes, with *K*_i_ values in the lower μM range. Except for DPA, none of the compounds showed significantly stronger inhibition of the virulence factors than of the human zinc metalloproteases. TPA and Zn230 were the only compounds that inhibited all five zinc metalloproteinases with a *K*_i_ value in the lower μM range. The thiophene compounds gave weak or no inhibition. Docking indicated that some of the compounds coordinated zinc by one oxygen atom from a hydroxyl or carbonyl group, or by oxygen atoms both from a hydroxyl group and a carbonyl group, and not by pyridine nitrogen as in DPA and TPA.

## 1. Introduction

Proteases (proteinases, peptidases, proteolytic enzymes) are involved in a variety of biological processes and comprise many enzymes in different families with structural and catalytic diversity. All proteases have a common ability to hydrolyze peptide bonds [1,2,3]. In humans, proteases are associated with different physiological processes including cellular signaling and growth, angiogenesis, blood pressure regulation, coagulation, intestinal absorption of nutrition, reproduction, wound repair, hemostasis, and homeostasis [3,4,5,6,7]. In microorganisms, proteases are involved in processes such as generation of nutrition, growth, survival, virulence, and formation of biofilm [8,9]. Dysregulation of human proteases may contribute to disease, and several human proteases are targets for therapeutic interventions [10,11,12], while several bacterial proteases are considered as important targets for the next generation of antibacterial drugs [10,13,14].

Metalloproteases are a heterogeneous group of proteases using a metal ion to bind the substrate and polarize a water molecule to perform the hydrolytic reaction. One of the human enzyme families that utilize a zinc-ion in the hydrolytic reactions is the matrix metalloproteases (MMPs). They belong to the M10 family of proteases [15], are calcium-dependent, and contain both a catalytic and a structural zinc ion [16]. The catalytic zinc is coordinated by three histidine residues and a water molecule in activated MMPs [17,18], and they are classified by the MEROPS database in the subclan metzincins [15]. MMPs are often overexpressed and functionally involved in a wide range of pathological conditions and are considered important drug targets [10,11,19,20,21,22]. However, the development of clinically relevant MMP inhibitors for therapeutic interventions has so far shown limited success, with a lack of efficacy and severe side effects in clinical trials [23,24]. More knowledge about the regulation of MMPs in different tissues and organs may be necessary so that compounds that promote positive effects and inhibit deleterious effects of specific MMPs for disease may be developed. In humans, there are 23 different MMPs. Most of them are secreted from cells as inactive proenzymes, while six are membrane-anchored through a type I transmembrane domain or a glycosyl-phosphatidyl-inosityl (GPI) moiety [16]. Membrane-anchored MMPs have the catalytic site extracellularly and are also called membrane-type metalloproteases (MT-MMPs). MMP-9 (gelatinase B) is the most studied secreted MMP, while MMP-14 (also called MT1-MMP) is the most studied MT-MMP. Several three dimensional (3D) structures of MMP-9 with and without inhibitors are known from X-ray crystallography [25], while 3D structures of MMP-14 in complex with a small molecular inhibitor are not published, but a complex of MMP-14 with the tissue inhibitor-2 of matrix metalloproteases (TIMP-2) is deposited in the PDB-database (PDB id: 1BQQ).

The zinc-metalloproteases thermolysin (TLN) from *bacillus thermoproteolyticus*, pseudolysin (PLN) from *pseudomonas aeruginosa* (LasB, or elastase of *P. aeruginosa*) and aureolysin (ALN) from *staphylococcus aureus* are secreted bacterial virulence factors that belong to the M4 family of proteases [13,15]. In the active enzymes, the catalytic zinc is coordinated by two histidines, a glutamic acid and a water molecule. In addition to the catalytic zinc, TLN has four calcium ions, while ALN has three and PLN has two. These enzymes are classified by the MEROPS database in the subclan gluzincins [15]. The secreted virulence factors degrade extracellular proteins and peptides of the host for bacterial nutrition and contribute to biofilm formation. The spread of antibiotic multi-resistance among central human pathogens is recognized by the World Health Organization (WHO) as a major global health concern and a pressing societal challenge, and new treatment options are needed to combat the spread [26]. Compounds inhibiting the virulence and not directly targeting the growth and viability may be an alternative treatment to present antibiotics, either alone or as an adjuvant to antibacterial drugs [27,28,29,30], and PLN, TLN and ALN are therefore interesting drug targets. The 3D structures of TLN and PLN were extensively studied with and without inhibitor [25], while only the 3D structure of the free enzyme is known for ALN (PDB id: 1BQB).

The ratio of the effect on virulence factors over effects on human zinc metalloproteases should be optimized to increase the therapeutic value of potential antibacterial drug candidates. The zinc metalloproteases cleave protein and peptide substrates at the N-terminal side of the amino acid occupying the S_1_’-subpocket. MMPs and virulence factors of the M4 family preferentially accept hydrophobic amino acids in the S_1_’-subpocket as shown in the MEROPS database [15], indicating structural similarities in their catalytic sites. X-ray structures of the enzymes show that the region around the catalytic zinc ion has structural similarities. Identifying potent inhibitors with high selectivity is therefore challenging.

Small molecules that inhibit zinc metalloproteases contain a chemical group that replaces the zinc coordinating water molecule in the catalytic site of the active enzymes and coordinates the zinc ion. Phosphinate (PO_2_^−^), carboxylate (COO^−^), thiolate (S^−^) and hydroxamic acid HONH-CO have shown to be effective zinc-binding groups leading to potent inhibition of zinc metalloproteases [31,32,33,34,35]. In solution, metal chelating groups with oxygen as donor atom are expected to be quite unselective for the chelating cation, while chelating groups with nitrogen as donor atom are expected to bind weaker to sodium, calcium, potassium, iron and manganese or other relevant biological cation than to zinc [36]. Compounds containing metal chelating groups with nitrogen as donor atom may therefore show selectivity for zinc enzymes in front of other metalloenzymes. Derivatives of the zinc chelators di(2-pyridinemethyl)amine (dipicolylamine, DPA) and tri(2-pyridinemethyl)amine (tripicolylamine, TPA) with nitrogen as putative donor atom were found to inhibit metallo-β-lactamases (MBLs) [37,38,39,40]. MBLs are a group of zinc-dependent hydrolytic enzymes responsible for breaking down β-lactam antibiotics, including carbapenems, penicillins and cephalosporins [41]. Their most likely mechanism of inhibition is by interference with zinc in the active site giving irreversible inhibition [42,43]. In the present study derivatives of DPA, TPA, tris pyridine ethylene diamine (TPED), pyridine and thiophene were tested for their inhibition of MMP-9 as a representative of secreted MMPs, and MMP-14 as a representative of MT-MMPs, and against the bacterial virulence factors TLN, PLN and ALN. 

## 2. Results and Discussion

Twenty TPA, DPA, TPED, pyridine and thiophene derivatives were studied using inhibition kinetics and molecular modeling. Some of the TPA and DPA analogs were previously shown to be slow or irreversible inhibitors of MBLs [38,39,40]. Therefore, we decided to test if the 20 compounds are time-dependent inhibitors of the bacterial virulence factors TLN, PLN and ALN and two human zinc metalloproteases, MMP-9 and MMP-14. In addition, we tested if TPA is a reversible or irreversible time-dependent inhibitor of MMP-14. The compounds were also tested for putative quenching of the fluorescence product formed during catalytic cleavage. The binding modes of the compounds in the five enzymes were studied by docking the compounds into the catalytic site of the five zinc metalloproteases.

### 2.1. Quenching 

Quenching experiments were performed with varying concentrations of the compounds (0–100 μM) against varying fluorescence product (McaPL-OH) concentrations as previously described for procaspase-activating compounds (PACs), isatin derivatives, bisphosphonate and catechol containing compounds [44,45]. Primary and secondary quenching plots revealed that none of the compounds quenched the fluorescence of the product. Most of the 20 compounds gave a background fluorescence at the wavelengths used, but this did not affect the inhibitory assays as enzymatic reactions were followed continuously. 

### 2.2. The Inhibition of MMP-14 by TPA

**Figure 1:**
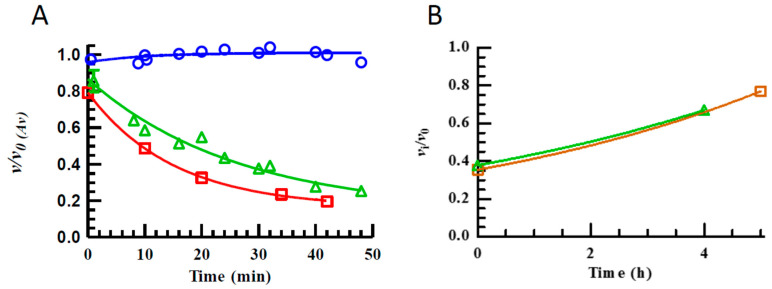
Time-dependent reversible inhibition of MMP-14 with TPA. (**A**): MMP-14 (45 (∆) and 5.6 (□) nM) was pre-incubated at 37 °C with 10 and 11.1 μM TPA, respectively, while controls were without TPA (○). Aliquots were withdrawn and the enzymatic activity determined. Initial rate velocities (*v*) at the different time points were divided by the average of the control velocities (*v*_0(Av)_). The results for the high concentration of MMP-14 in the pre-incubation assay are from two independent experiments. (**B**): Two independent experiments (Δ and □) of micro-dialysis (4 °C) of MMP-14 (45 nM) pre-incubated at 37 °C. Aliquots were withdrawn after various time points and the initial rate velocities determined. The velocity ratio *v*_i_/*v*_0_ was determined for each time point where *v*_i_ is the initial rate of the MMP-14/TPA sample and *v*_0_ of the MMP-14 control at the same dialysis time point.

We tested if TPA is a time-dependent irreversible or reversible inhibitor of MMP-14. In these experiments, two different concentrations of MMP-14 (45 and 5.6 nM) were pre-incubated up to 48 min at 37 °C with 10.0 and 11.1 μM of TPA, respectively, as described in Materials and Methods. As controls, the same amount of MMP-14 (without TPA but with the same concentration of DMSO) was identically pre-incubated at 37 °C. In the activity assay (10 μM of the substrate McaPLGL(Dpa)AR-NH_2_) the pre-incubation mixture with or without TPA and with a high concentration of MMP-14 was diluted 10 times which resulted in a TPA concentration in the activity assay of 1 μM, while the mixture with the low concentration of MMP-14 was only diluted 1.1 times, and hence the TPA concentration in the activity assay was 10 μM. Figure 1A shows that the MMP-14 control without inhibitor present was stable during the entire pre-incubation period, while the presence of TPA resulted in a time-dependent inhibition of the enzyme. In the presence of 100 μM TPA, *v*_i_/*v*_0_ was 0.61 after 2 min and 0.03 after 11 min pre-incubation at 37 °C. To determine whether the observed time-dependent inhibition was due to reversible or irreversible inhibition, a solution of 45 nM MMP-14 was first pre-incubated for approximately 40 min at 37 °C with 10 μM TPA (1% DMSO in assay buffer, pH 7.5). As a control, MMP-14 (without TPA but with the same concentration of DMSO) was also pre-incubated for approximately 40 min at 37 °C. Thereafter, aliquots (20 or 30 μL) of MMP-14 control and MMP-14/TPA mixture were applied to micro-dialysis at 4 °C as described in Materials and Methods. Figure 1B shows results from two representative independent micro-dialysis experiments. The activity (*v*_i_/*v*_0_) increased after dialysis, and hence TPA is slowly released from the MMP-14/TPA complex. This indicates that TPA is a slow, reversible inhibitor of MMP-14, and therefore we assume the tested TPA, DPA, TPED, pyridine and thiophene derivatives most probably are not inactivators/irreversible inhibitors of the tested zinc metalloproteinases, in contrast to the previous observations for MBLs [38,39,40].

### 2.3. Inhibitory Effects

**Figure 2:**
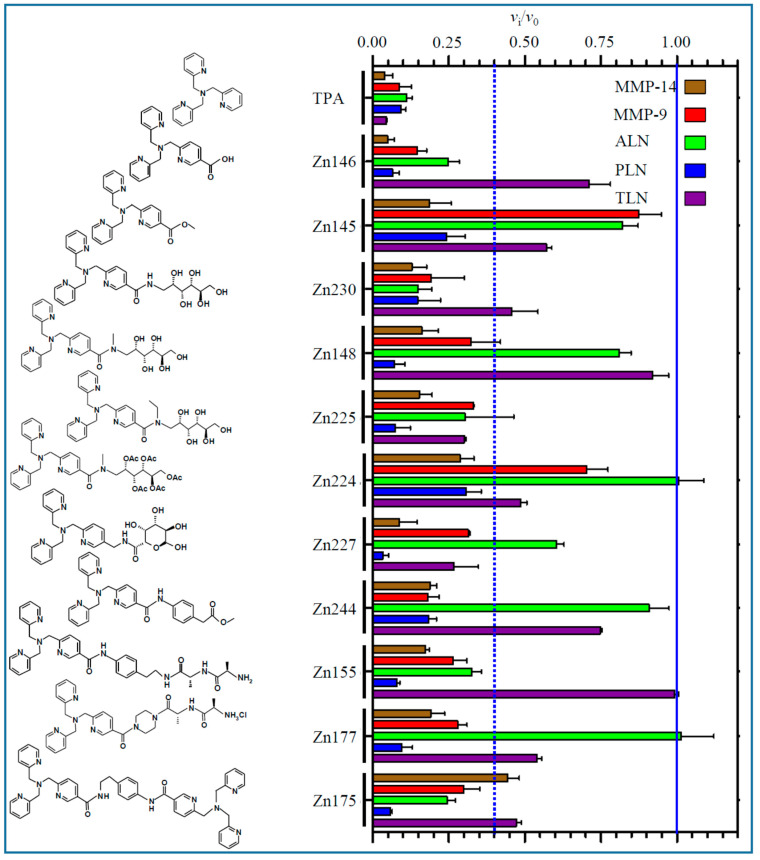
Inhibitory effect of 100 μM of TPA derivatives on the activity of MMP-9, MMP-14, TLN, PLN and ALN. For compounds with fast inhibitory binding, the *v*_i_/*v*_0_ (mean ± s.e.m.) was based on the mean values after 0–45 or 0–30 min pre-incubation. For compounds with time-dependent slow inhibition the *v*_i_/*v*_0_ (mean ± s.e.m.) was based on pre-incubation time points where the time curve in supplementary material Figure S1 flattens out. For compounds where the time curve was not flattening out after 45 min of pre-incubation (not reached equilibrium), the mean value was for 30 and 45 min pre-incubation.

**Figure 3:**
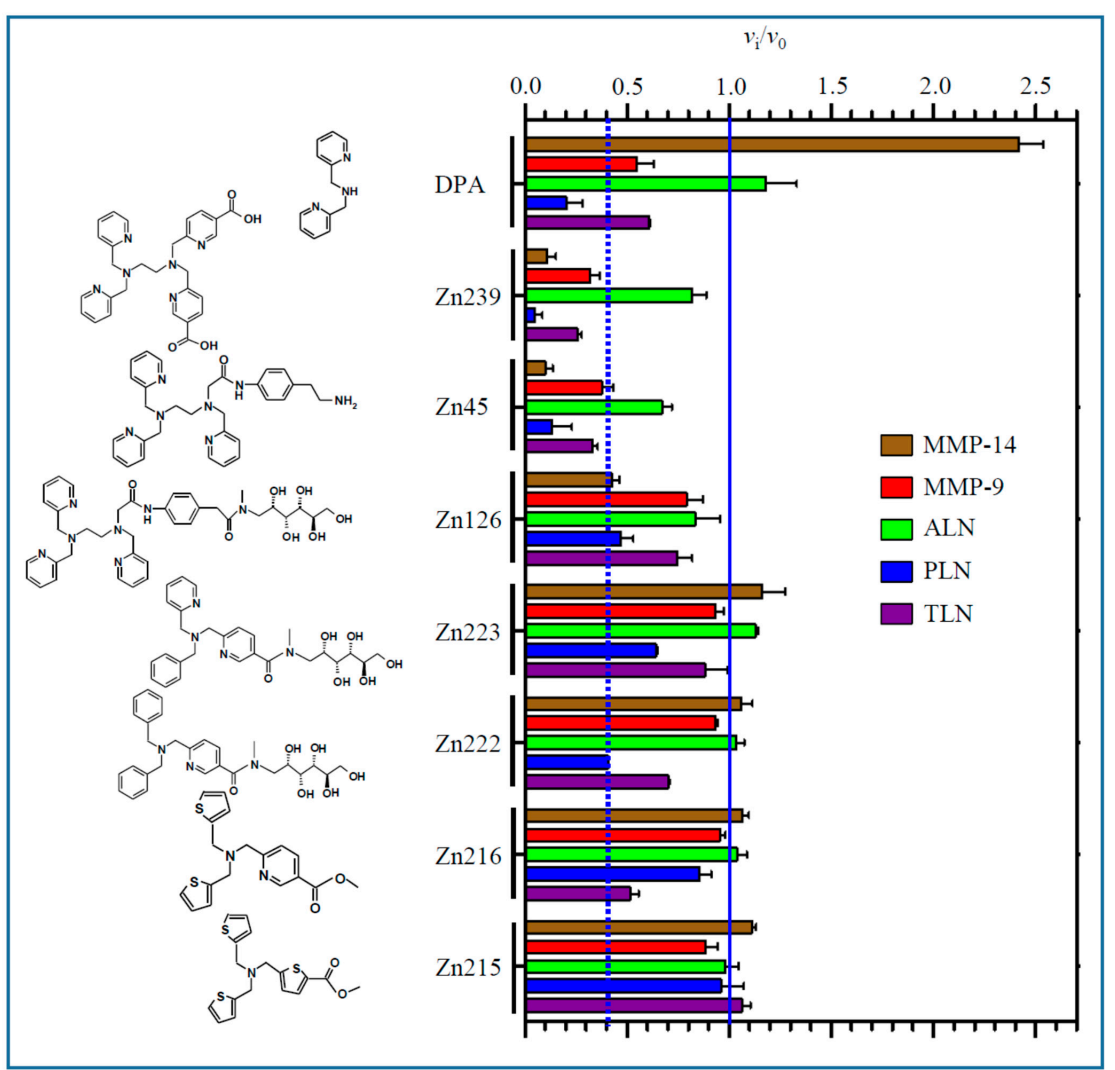
Inhibitory effect of 100 μM of DPA, TPEN, pyridine and thiophene derivatives on the activity of MMP-9, MMP-14, TLN, PLN and ALN. For compounds with fast inhibitory binding, the *v*_i_/*v*_0_ (mean ± s.e.m.) was based on the mean values after 0–45 or 0–30 min pre-incubation. For compounds with time-dependent slow inhibition, the *v*_i_/*v*_0_ (mean ± s.e.m.) was based on the pre-incubation time points where the time curve in Figure S2 flattens out. For compounds where the time curve was not flattening out after 45 min of pre-incubation (not reached equilibrium), the mean value was for 30 and 45 min pre-incubation.

A total of 100 μM of the compounds was either pre-incubated with the protease for 15, 30 and 45 min at room temperature and the enzymatic reaction was started by adding the substrate, or not pre-incubated, i.e., the reaction was started by adding the protease to a mixture of substrate and compound as described in Materials and Methods. The controls were performed in the same way but without inhibitor present. Figure 2 and Figure 3 show the relative binding strength at 100 μM of the 20 compounds to MMP-9, MMP-14, ALN, PLN and TLN. For compounds showing fast inhibition, each bar represents the mean of the results after 0–30 or 0–45 min pre-incubation. When time-dependent/slow inhibition was seen, the bars represent the mean of time points after the time curve has flattened out, and the slope of the curve is approximately zero (enzyme and inhibitors have reached an equilibrium) (supplementary material, Figures S1 and S2). For compounds where the time curve was still decreasing after 45 min pre-incubation, the mean of the 30 and 45 min pre-incubation time was used to give an impression of the relative binding strength of the compound.

As shown for some of the compounds in supplementary material Figures S1 and S2, the same compound had completely different effects on the five zinc metalloproteases. One compound that did not give any effect on the activity of one enzyme, could be a fast binding inhibitor with different binding strengths on other enzymes and be a slow and time-dependent inhibitor of another. One of the compounds (DPA) had a surprising effect on MMP-14, where the rate of the enzymatic substrate cleavage was greatly enhanced at all the different incubation time points (supplementary material Figure S2). The mechanism behind this was not further investigated. For most compounds with time-dependent inhibition, the inhibitory *v*_i_/*v*_0_ curve was flattening out after 15 or 30 min, but for a few compounds the curve did not flatten out, indicating that the enzyme/inhibitor complex had not reached an equilibrium (supplementary material, Figures S1 and S2). The reason might be a very slow, reversible binding. This was not further investigated.

**Figure 4:**
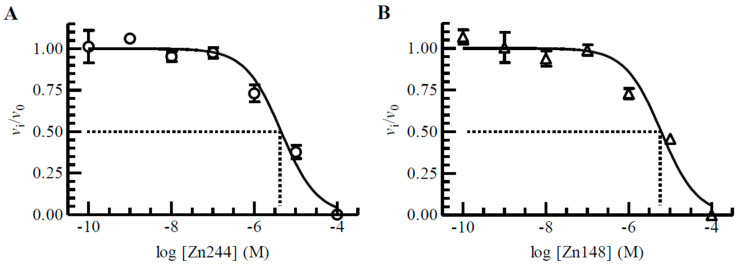
Dose–response plots of Zn244 (**A**) and Zn148 (**B**) for PLN. The enzyme with and without inhibitor was pre-incubated for 30 min at room temperature and the reaction started by adding McaRPPGFSAFK(Dnp)-OH (4 μM in assay), where *v_i_* and *v_0_* are initial reaction rates in the presence and absence of Zn244 and Zn148. Each point on the curve shows the mean ± s.d. (N = 3 for all points in (**A**) and N = 4 for all points in (**B**) with regression coefficients r^2^ of 0.97 in (**A**) and 0.95 in (**B**), giving an *IC*_50_ value of 4.46 ± 0.05 μM and a *K*_i_ value of 4 ± 1 μM for Zn244 (**A**) and an *IC*_50_ value of 6.07 ± 0.08 μM and a *K*_i_ value of 5 ± 2 μM for Zn148 (**B**). The large s.d. of the *K*_i_ values compared to the *IC*_50_ values is due to the large s.d. in the *K*_m_ value of the substrate in absence of an inhibitor.

When 100 μM of the test compound gave an inhibition of 60% or more (*v*_i_/*v*_0_ ≤ 0.4) at 30 min pre-incubation, further experiments were performed to obtain dose–response curves and *K*_i_ values calculated from the obtained *IC*_50_ values. To ensure comparable results, all *IC*_50_ and *K*_i_ values were based on 30 min pre-incubation of the enzyme and tested compound. Figure 4A,B show typical dose–response curves for Zn244 and Zn148 against PLN. Equation (1) in the Materials and Methods section was used to calculate *IC*_50_ values. 

For calculating *K*_i_ values from the obtained *IC*_50_ values it is necessary to know the *K*_m_ value for the enzyme with the substrate at the conditions in the inhibition experiments. Equation (2) in Materials and Methods shows the relation between *K*_i_ and *IC*_50_ values for a competitive inhibitor. *K*_m_ values for the enzymes at the conditions used in the present work were determined previously [44]. To summarize, the *K*_m_ values of the substrate McaPLGL(Dpa)AR-NH_2_ with MMP-9 and MMP-14 were 4 ± 1, and 4.9 ± 0.4 µM, respectively, while the *K*_m_ values of the substrate McaRPPGFSAFK(Dnp)-OH with ALN, PLN and TLN were 76 ± 7, 24 ± 8 and 6 ± 1 µM, respectively. Estimated *K*_m_ values for ALN and PLN must be regarded as uncertain since the highest substrate concentration was 10 µM due to quenching. Table 1 shows the obtained *K*_i_ values. None of the compounds were tight binders, i.e., *K*_i_ values close to or less than the enzyme concentration in the assay. Most of the compounds showed inhibition of PLN and MMP-14 in the lower μM region, while only 30–50% of the compounds had *K*_i_ values in the lower μM region for MMP-9, TLN and ALN.

None of the compounds showed selective inhibition favoring bacterial zinc metalloproteases over the two human MMPs, but DPA inhibited PLN much stronger than the other enzymes. In general, the compounds showed quite similar inhibition of PLN and MMP-14, and in general, the *K*_i_ values for these enzymes were lower than for the other enzymes (Table 1).

The TPA derivative Zn148 (*K*_d_ (zinc) = 10^−12^ M), which is an irreversible inhibitor of the MBLs VIM-2 and NDM-1, was shown to reverse carbapenem resistance in Gram-negative pathogens in vivo without acute toxicity in female BALB/c mice [39]. These authors also showed that Zn148 derivatives with reduced zinc-binding, Zn222 (*K*_d_ (zinc) = 10^−6^ M) and Zn223 (*K*_d_ (zinc) = 10^−7^ M), lacked the ability to reverse carbapenem resistance in Gram-negative pathogens. The former compound did not inactivate the two MBLs, while the latter showed a reduced capability to inactivate the two MBLs compared to Zn148. The present results show that Zn148 is a decent inhibitor of the PLN and MMP-14, while 100 μM of the compound had no or only a limited effect on the other two bacterial proteases (Table 1, Figure 2, supplementary material Figure S1). This compound reacts slower with human MMP-9 than with MMP-14 and PLN, and the time curve did not flatten out after 45 min (supplementary material, Figure S1). It was also notable that the two compounds where one or two pyridine groups were replaced by a benzene ring (Zn223 and Zn222) resulted in weaker binding than Zn148 to PLN, MMP-14 and MMP-9, and no effect on TLN and ALN (Figure 3 and Figure S2). 

Although our results suggest that the TPA derivatives are not irreversible inhibitors of the five zinc metalloproteases, there are similarities with their effects on MBLs, as the strongest zinc chelator binds strongest to both the proteases and the MBLs. PLN, the elastase of *P. aeruginosa* is known to play a pivotal role in pseudomonal infections, and the inhibition of PLN by Zn148 (*K*_i_ of 5 μM) suggests that this inhibition may strengthen the potential of Zn148 as an antibacterial adjuvant against *P. aeruginosa* infections. In most human tissues, MMP-14 is constitutively expressed while MMP-9 is induced, and both are involved in several physiological processes. The present results encourage further investigations into off-target effects of this class of putative antibacterial adjuvants [39]. 

### 2.4. Enzyme Interactions

**Figure 5:**
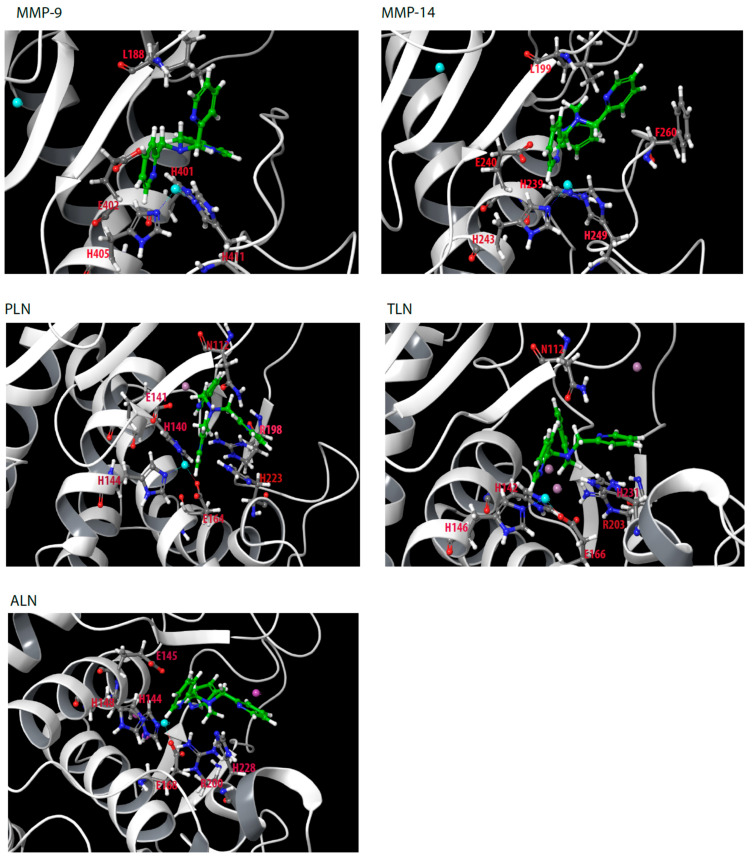
TPA docked into the catalytic site of MMP-9, MMP-14, PLN, TLN and ALN. Some of the most important amino acids for TPA binding are shown. Color coding of amino acids: carbon; grey, oxygen; red, nitrogen; dark blue, hydrogen; white. Color coding of TPA: carbon; green, nitrogen; blue, hydrogen; white Color coding of ions: zinc; light blue sphere, calcium; pink sphere.

Of the 20 compounds, only TPA and Zn230 showed strong binding to all five proteases (Figure 1, Table 1). In solution, TPA has a *K*_d_ for zinc of approximately 10^−11^ M [40,46]. Docking showed that the binding mode of TPA was quite similar in all five zinc metalloproteases (Figure 5). In PLN, the nitrogen atom in one of the pyridine rings coordinated zinc. His223 formed a hydrogen bond with the same nitrogen atom and had pi–pi stacking interactions with the pyridine ring. Another pyridine ring interacted within the S_1_’-subpocket forming hydrophobic interactions with the side chains of Leu132, Val137 and Ile190. This pyridine ring also formed pi–pi stacking interactions with His140 and pi–cation interactions with Arg198. The third pyridine ring was in the direction of the S_1_-subpocket, being more exposed to solvent than the other pyridine rings. In addition, this pyridine ring formed pi–pi stacking interactions with His223 and pi–cation interactions with Arg198. The amine nitrogen atom interacted with Glu141 and Asn112.

**Figure 6:**
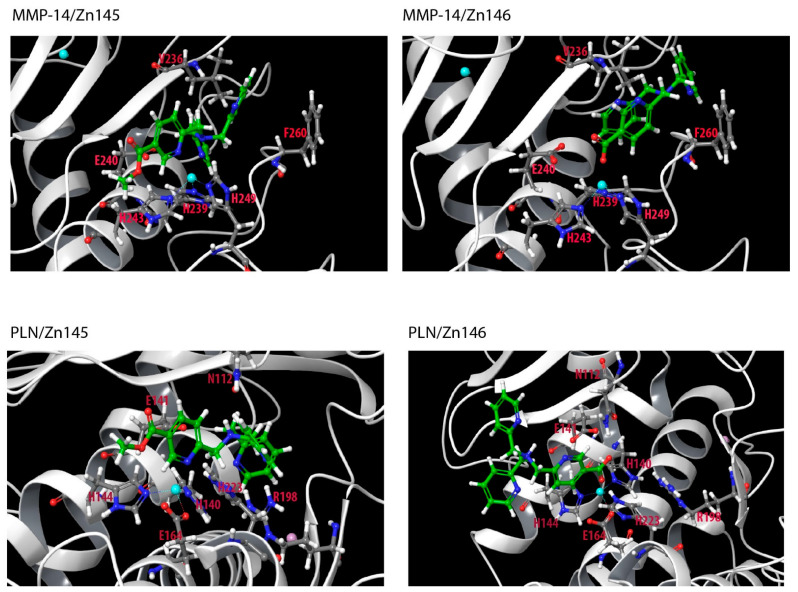
Zn145 and Zn146 docked into the catalytic site of MMP-14 and PLN. Some of the most important amino acids for binding Zn145 and Zn146 are shown. Color coding as in Figure 5.

The introduction of a carboxylic acid functionality (Zn146) and its methyl ester (Zn145) reduced the binding to MMP-9, TLN and ALN compared with TPA, but not to MMP-14 and PLN (Figure 2, Table 1). Docking of Zn146 showed that the carboxylic acid was responsible for coordinating the zinc in all enzymes, and not any of the pyridine nitrogen atoms (Figure 6). In PLN, one pyridine ring was into the S_1_-subpocket forming pi–pi stacking interactions with Tyr114, while another was exposed to solvent. Similar binding modes were also seen in the other enzymes. In MMP-14 pi–pi stacking interactions were formed with Phe198 (S_1_’-subpocket), in addition to a hydrogen bond between pyridine nitrogen and Leu199 (S_1_’-subpocket), for one of the pyridine rings. Zn239 contains two carboxylic acid groups (Figure 3), and docking indicated that the carboxylic acid group not interacting with zinc, interacted with Glu111 in PLN, while one pyridine ring had pi–pi stacking with Tyr155 (S_1_-subpocket), another with His223, while one pyridine nitrogen formed a hydrogen bond with Asn112. Docking of Zn145 showed that the nitrogen atom of the pyridine ring containing the methyl ester functionality ligated zinc, while the methyl ester group was exposed to solvent in both PLN and MMP-14 (Figure 6). However, in PLN docking poses of Zn145 with the methyl ester group interacting with Arg198 were also obtained during the docking experiments, but still, the pyridine nitrogen coordinated zinc. MMP-14 is lacking an arginine corresponding to Arg198 in PLN, and that may be the reason that only one orientation of Zn145 was obtained with MMP-14. The reason for zinc coordination by pyridine nitrogen in Zn145 that contains the carboxylic acid ester functionality may be that the carboxylic acid ester is less polar and bulkier than the carboxylic acid functionality in Zn146 giving steric hindrances.

**Figure 7:**
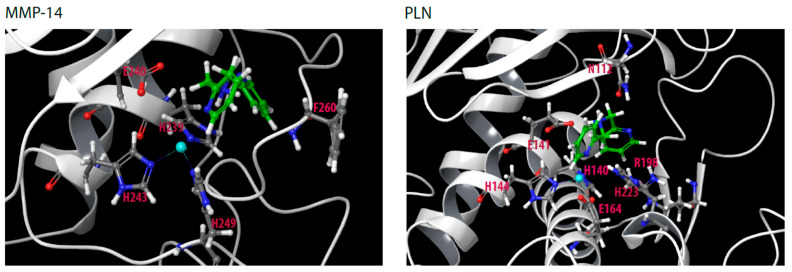
Docking of DPA into the catalytic site of MMP-14 and PLN. Some of the most important amino acids for binding DPA are shown. Color coding as in Figure 5.

Removal of one 2-methylpyridine group of TPA gives DPA. DPA is a weaker zinc-binder (*K*_d_ ~ 10^−8^ M) than TPA (*K*_d_ ~ 10^−11^ M) [37,46], and its inhibition of the five proteases was largely reduced compared to TPA, except for PLN where DPA and TPA were equally effective (Figure 2 and Figure 3, Table 1). Moderately strong inhibition of DPA was only seen for PLN (Figure 3, Table 1). Docking of DPA into PLN showed that pyridine nitrogen coordinated the zinc. The zinc interacting pyridine ring also had pi–pi stacking interactions with His223. The other pyridine ring interacted with hydrophobic amino acids (Val137, Ile186, Gly187, Ile190) and Asp136 in the S_1_’-subpocket and formed pi–pi stacking interactions with the zinc ligated amino acid His140. In addition, the amine nitrogen atom interacted with the catalytic amino acid Glu141 (Figure 7). Similar binding modes were also seen in TLN and MMP-9. As previously mentioned, DPA increased the rate of the substrate cleavage by MMP-14 at all pre-incubation time points (Supplementary, Figure S2). Interestingly, docking into MMP-14 indicated a binding mode different from that in PLN. The docking program did not recognize any clear zinc binding, and the distance between zinc and the closest pyridine nitrogen was approximately 3 Å in the highest scored docking pose, while the corresponding distance in PLN was approximately 2 Å. The pyridine ring containing this nitrogen atom had pi–pi stacking interactions with the zinc ligated amino acid His239 (as in PLN). In addition, an amine hydrogen atom formed a pi–cation interaction with His239 and a hydrogen bond with Pro259 (S_1_’-subpocket), while the other pyridine ring interacted with Tyr261 (pi–pi stacking) in the S_1_’-subpocket. The binding mode indicated that the substrate may interact within the substrate binding cleft despite bound DPA. A putative explanation for the increased rate of substrate cleavage may be that the position of the pyridine ring closest to zinc may contribute to a more favorable binding mode of the substrate McaPLGL(Dpa)AR-NH_2_ for cleavage. 

Putative scaffolds for zinc chelation in most of the compounds are the DPA or TPA molecules, with nitrogen as the expected donor atom for zinc coordination. However, during docking, the derivatives containing carboxylic acid (Zn146 and Zn239) coordinated zinc via the carboxylic acid. Zn230, Zn148 and Zn225 have the hydrophilic methyl-amino glycosyl side chain linked to the TPA molecule via an amide bond that is either unmodified (Zn230), methylated (Zn148) or ethylated (Zn225). Except for PLN, reduced inhibition relative to TPA was obtained for these derivatives, but their difference in inhibition of PLN and MMP-14 was small (Figure 2, Table 1). Derivatives with a structural change of one of the 2-methylpyridine groups of TPA had little effect on the enzyme activity of MMP-14 and PLN. That may suggest that their main interactions with PLN and MMP-14 are mainly through the 2-methylpyridine groups. However, this suggestion is contradictory to the docking results that indicated that, in addition to Zn146 and Zn239, derivatives containing the methyl-amino glycosyl side chain (Zn230, Zn148 and Zn225, Zn126) also coordinated by one or more oxygen atoms, either via one hydroxyl group, the carbonyl group, or both a hydroxyl group and the carbonyl group. Docking indicated that all other TPA and DPA derivatives, including those containing a carboxylic acid ester functionality (Zn145, Zn244) coordinated zinc by pyridine nitrogen. The exception was Zn45 (Figure 2), which coordinated zinc by the carbonyl group.

**Figure 8:**
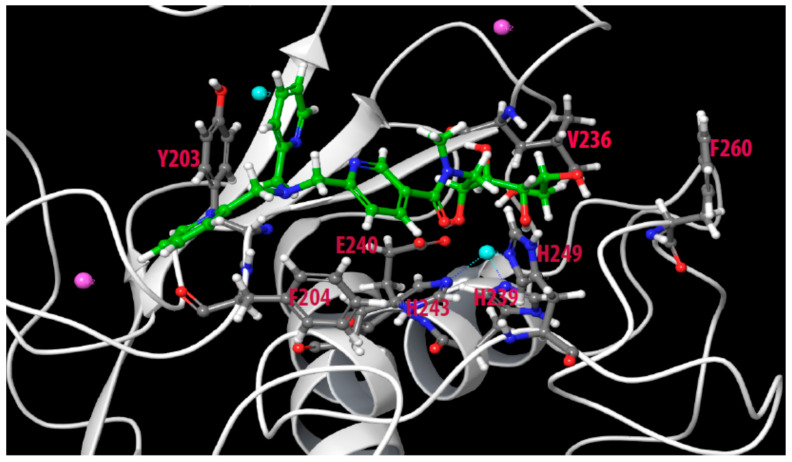
Docking of Zn148 into the catalytic site of MMP-14. Some of the most important amino acids for binding Zn148 are shown. Color coding as in Figure 5.

An interesting feature was when one or both the two unmodified 2-methylpyridine groups of Zn148 were replaced by a benzylic group (Zn223 and Zn222) the MMP-14 binding was prevented compared with Zn148 (Figure 2 and Figure 3). This suggests that the nitrogen in both these 2-methylpyridine groups is needed for MMP-14 binding. Similarly, these two modifications weaken but do not prevent the binding to PLN. The importance of the two unmodified 2-methylpyridine groups for the binding to MMP-14 and PLN is also shown by the weak inhibition or lack of inhibition by the two compounds Zn216 and Zn215, where the 2-methylpyridine groups are replaced by 2-methylthiophene groups. That may suggest that the pyridine nitrogen atoms of Zn148 are directly involved in binding or that the electron distribution of the pyridine rings is important. Docking of Zn148 into MMP-14 (Figure 8) showed that the zinc-binding was both by the carbonyl group and a hydroxyl group, and not by pyridine nitrogen. However, the pyridine rings were still important for interactions, as one pyridine nitrogen interacted with Phe204 in the S_1_-subpocket, while Phe204 also formed pi–pi stacking interactions with another pyridine ring, while the third pyridine ring formed pi–pi stacking with Tyr203. The sugar chain also formed hydrogen bonds with the catalytic residue Glu240 and with Ala200 (S_1_’-subpocket) and Pro259 (S_1_’-subpocket). 

## 3. Materials and Methods

### 3.1. Materials

Dimethyl sulfoxide (DMSO), TRIS, sodium hydrogen phosphate (Na_2_HPO_4_)**,** and sodium acetate (CH_3_COONa) were from Merck (Darmstadt, Germany). Ethylenediaminetetraacetic acid was from Fluka (Buchs, Switzerland). Acrylamide, Coomassie Brilliant Blue G-250 and Triton X-100 were from BDH (Poole, UK). Hepes, Brij-35, Silver nitrate, alkaline phosphatase-conjugated antibodies and gelatin were from Sigma (St. Louis, MO, USA). Gelatin-Sepharose, was from GE-Healthcare (Uppsala, Sweden). DC Protein Assay and unlabeled molecular weight standards were from BioRad (Richmond, CA, USA). Sf9 insect cells and Magic Marker molecular weight standards were from Invitrogen (Carlsbad, CA, USA). Western Blotting Luminol reagent and HRP-conjugated donkey anti-goat secondary antibody were from Sancta Cruz (Santa Cruz, CA, USA). HRP-conjugated goat anti-rabbit secondary antibody was from Southern Biotech (Birmingham, AL, USA). Fetal bovine serum was from Biochrom AG (Berlin, Germany). Galardin (Gm6001), human MT1-MMP/MMP-14 (catalytic domain), TLN and PLN were from Calbiochem (San Diego, CA, USA) and Aureolysin was from BioCentrum Ltd. (Kraków, Poland). McaPLGL(Dpa)AR-NH_2_ (ES001) and McaRPPGFSAFK(Dnp)-OH (ES005) were from R&D Systems (Minneapolis, MN, USA). 

### 3.2. Compounds for Testing

Twenty TPA, DPA, TPEN, pyridine and thiophene derivatives were tested as putative inhibitors of MMP-9, MMP-14, PLN, TLN and ALN. Synthesis of the compounds was reported previously [37,38,39,40]. The structure of the compounds is shown in Figure 2 and Figure 3.

### 3.3. Expression, Purification and Activation of Recombinant Human proMMP-9 in Sf9 Insect Cells

The expression and purification of recombinant human full-length proMMP-9 (rpMMP-9) from Sf9 insect cells were performed as described previously [47]. The amount of proMMP-9 was estimated spectrophotometrically at 280 nm using ε_280_ nm = 114.36 mM^−1^ cm^−1^ [48]. Activation of the recombinant proMMP-9 was performed with APMA (auto-activation) as described previously [47]. The amount of active MMP-9 was determined by active site titration using galardin also described previously [47].

### 3.4. Determination of Reaction Velocities

McaPLGL(Dpa)AR-NH_2_ was used as a substrate for MMP-9 and MMP-14, while McaRPPGFSAFK(Dnp)-OH was used as substrate for ALN, PLN and TLN. The reaction velocities/initial rates (*v*) were determined at 37 °C, at an excitation wavelength of 320 nm and an emission wavelength of 405 nm with a slit width of 10 nm using either a Perkin Elmer LS 50 Luminescence spectrometer and the FL WinLab Software Package (Perkin Elmer) or a Clario Star microplate reader (CLARIOstar^®^ BMG LABTECH).

The test compounds were dissolved in 100% DMSO giving a concentration of 10 mM. A fixed substrate concentration of 4.0 µM in a total volume of 100 µL 0.1 M Hepes pH 7.5, 10 mM CaCl_2_, 0.005% Brij-35 and 1.0% DMSO, were used in all assays, except for ALN where the substrate concentration was 5.0 µM. The fixed enzyme concentrations were as follows; 0.05 nM MMP-9, 1.0 nM MMP-14, 1.4 nM ALN, 0.5 nM PLN and 0.21 nM TLN. Time-dependent inhibitory experiments without and with 100 μM of testing compounds were performed as follows. Compounds were either pre-incubated with the proteases for 15, 30 and 45 min at room temperature and the enzymatic reaction was started by adding the substrate, or not pre-incubated, i.e., the reaction was started by adding the protease to the mixture of substrate and compound. Control tests without compound present were performed in the same way. The reaction was followed for 30 min at 37 °C.

### 3.5. Testing of Slow, Reversible/Irreversible Binding of TPA

In pre-incubation tests with high concentration of MMP-14, 90 μL 50 nM MMP-14 was mixed with 9.0 μL 110 μM TPA (dissolved in 0.1 M Hepes pH 7.5 containing 10 mM CaCl_2_, 0.005% Brij-35 and 10% DMSO) and incubated at 37 °C for up to 48 min. As a control, 90 μL 50 nM MMP-14 was mixed with 9.0 μL 0.1 M Hepes pH 7.5 containing 10 mM CaCl_2_, 0.005% Brij-35 and 10% DMSO. At different time points, 10 μL of the enzyme (with and without TPA) was added to 90 μL of 0.1 M Hepes pH 7.5 containing 10 mM CaCl_2_, 0.005% Brij-35 and 11.1 μM ES001 and the initial rate reaction velocity (*v*) was determined as described in Section 3.4. using a Perkin Elmer LS 50 Luminescence spectrometer. The reaction was followed for 1 min (10 data points collected per second). The TPA concentration was 10 μM in the pre-incubation assay and 1.0 μM in the reaction rate assay. 

In pre-incubation tests with a low concentration of MMP-14, 60 μL 50 nM MMP-14 was mixed with 420 μL 0.1 M Hepes pH 7.5 (containing 10 mM CaCl_2_, 0.005% Brij-35) and 60 μL of either 0.1 M Hepes pH 7.5 containing 10 mM CaCl_2_, 0.005% Brij-35 and 10% DMSO (control) or 60 μL 100 μM TPA (dissolved in 0.1 M Hepes pH 7.5 containing 10 mM CaCl_2_, 0.005% Brij-35 and 10% DMSO). At different time points, 90 μL of the enzyme (with and without TPA) was mixed with 10 μL 100 μM ES001 and the initial rate of the reaction was determined as described above. The TPA concentration is 11.1 μM in the pre-incubation assay and 10 μM in the reaction rate assay.

### 3.6. Microdialysis

Ninety μL of 50 nM MMP-14 was mixed with 9.0 μL of either 0.1 M Hepes pH 7.5 containing 10 mM CaCl_2_, 0.005% Brij-35 and 10% DMSO (control) or 110 μM TPA (dissolved in 0.1 M Hepes pH 7.5 containing 10 mM CaCl_2_, 0.005% Brij-35 and 10% DMSO) and incubated at 37 °C for approximately 40 min. After 0 and 40 min pre-incubation, 10 μL of an enzyme (with and without TPA) was mixed with 90 μL of 0.1 M Hepes pH 7.5 containing 10 mM CaCl_2_, 0.005% Brij-35 and 11.1 μM ES001 and the reaction velocity was determined as described above (Section 3.5). Samples pre-incubated for 40 min at 37 °C were added to microdialysis at 4 °C. For that, 1 mL Eppendorf pipette tips were cut and sealed with a dialysis membrane (cut-off 14 kDa) that were boiled and rinsed in Milli-Q water. Twenty or 30 μL of the sample (with and without TPA) was added to each tip and samples with and without TPA were dialyzed for up to 4 or 5 h in 100 mL of 50 mM Hepes pH 7.5 containing 5 mM CaCl_2_ and 0.005% Brij-35. After dialysis, the sample (with and without TPA) in the tip was removed and the total volume of the dialyzed sample was determined. If a sample at a time point contained more than added (i.e., >20 or 30 μL), the dilution was determined and compared with the other sample at that time point. At each time point, the initial rate was determined for an equal amount of enzyme (with and without TPA) based on the dilution factor. The reaction rate was determined as described above, where 10 μL sample (after correct dilution) was added to 90 μL of 0.1 M Hepes pH 7.5 containing 10 mM CaCl_2_, 0.005% Brij-35 and 11.1 μM ES001.

### 3.7. Determination of IC_50_ and K_i_ Values

The inhibitory constant *IC*_50_ of the various compounds were performed with concentrations ranging from 10^−10^ to 10^−4^ M in the assay, with a fixed substrate, enzyme and buffer concentration as described above. Enzymes with and without inhibitor were pre-incubated for 30 min at room temperature, and initial rate assays were performed as described above. Assays were performed using a Clario Star microplate reader (CLARIOstar^®^ BMG LABTECH). The *IC*_50_ values were calculated in Graph Pad Prism 5 using Equation (1):

(1) $$\frac{v_{i}}{v_{0}}=\frac{1}{\left(1+10^{\left(pIC50-pI\right)}\right)}$$

where *v*_i_ is the enzyme activity in the presence of inhibitor, *v*_0_ the activity in the absence of inhibitor, *pI* = −log [Inhibitor] in M and *pIC*_50_ = −log *IC*_50_ in M. All experiments were performed in at least triplicate.

Equation (2) shows the relation between *IC*_50_ and *K*_i_ values for substrate competitive inhibitors based on the used fixed substrate concentration and the enzymes *K*_m_ value for the substrate:

*IC*_50_ = *K*_i_ (1 + [S]/*K*_m_) (2)

### 3.8. Quenching Experiments

To determine to which extent the compounds quench the time-dependent enzymatic increase in the fluorescence product of the processed substrate, quenching experiments were performed as described previously [45]. Briefly, the fluorescence (λ_ex_ = 320 nm, λ_em_ = 405 nm, slit width = 10 nm) of various concentrations of the fluorescent product McaPL-OH (0–100 nM) of the substrate McaPLGL(Dpa)AR-NH_2_ was determined in the absence and presence of various concentrations of the test compounds (0–100 µM). Primary and secondary plots were used to determine whether these compounds quenched the McaPL-OH fluorescence.

### 3.9. Docking

The following enzyme structures in the PDB-database were used for docking: TLN; 5DPE, PLN; 1U4G, ALN; 1BQB, MMP-14; 1BQQ, MMP-9; 5CUH. All five X-ray structures were optimized using the Protein preparation Wizard in Schrodinger Suites (version 2021-1) with default setting [49]. Water molecules at the binding site were deleted. Overlapping atoms were corrected using restrained minimization with the OPLS4 force field [50]. Grid maps were generated for each structure with a van der Waals radius scaling factor of 1 Å and a partial cut-off of 0.25 Å. The co-crystalized ligands were used as the centroid of the grid map generation for PLN, TLN and MMP-9, while the catalytic zinc atom was the centroid for ALN and MMP-14. The 20 selected derivatives were prepared with the Ligprep program, using ionization states in the pH range of 7.4 ± 0.2, generation of tautomers and retaining the specified chirality. The compounds were docked into the five enzyme structures using Glide Standard Precision (SP) docking with enhanced sampling and generation of maximum 10 poses per compound. The docking result was ranked using docking score. Docking of the compounds was performed in triplicate. The best results were determined using both docking score and ligand orientation in the binding site.

## 4. Conclusions

Inhibition of bacterial virulence is believed to be a new treatment option for bacterial infections. Here we tested DPA, TPA, and derivatives of DPA, TPA, TPEN, pyridine and thiophene as putative inhibitors of the bacterial virulence factors TLN, PLN and ALN, the human zinc metalloproteases MMP-9 and MMP-14. The compounds showed stronger inhibition of PLN and MMP-14 than of the other enzymes, with *K*_i_ values in the lower μM range. TPA and Zn230 were the only compounds that inhibited all five zinc metalloproteinases with a *K*_i_ value in the lower μM range. Docking studies indicated that Zn146 and Zn239 coordinated zinc by the carboxylic acid functionality, while derivatives containing the methyl-amino glycosyl side chain (Zn230, Zn148 and Zn225, Zn126) also coordinated by one or more oxygen atoms, either via one hydroxyl group, the carbonyl group, or both a hydroxyl group and the carbonyl group. The other compounds coordinated zinc by pyridine nitrogen. Our studies indicated that TPA is a slow, reversible inhibitor of MMP-14, and it is therefore reasonable to believe that the tested compounds are not inactivators/irreversible inhibitors of the tested zinc metalloproteinases, in contrast to the previous observations for MBLs [38,39,40].

Several of the tested compounds including Zn148 were previously found to irreversibly inhibit MBLs by chelating the catalytic zinc and were suggested to be promising as adjuvants to antibacterial treatment with β-lactam antibiotics. Zn148 (Figure 2) was also found to reverse the carbapenem resistance in Gram-negative pathogens in vivo with limited in vivo toxicity [39]. PLN is the key zinc metalloproteinase virulence factor secreted by the opportunistic Gram-negative pathogen *P. aeruginosa*, which is characterized by the WHO as a critical pathogen for which new treatment options are urgently needed. The reversible inhibition of PLN by Zn148 and other compounds in the present study indicates that they may inhibit *P. aeruginosa* virulence and have potential as adjuvants in the treatment of pseudomonas infections.

## Figures and Tables

**Table 1:** *K*_i_ values of the TPA, DPA and TPED derivatives for MMP-14, MMP-9, ALN, PLN and TLN. *K*_i_ values determined for both bacterial and human metalloproteases using two different fluorescence quenched peptide substrates, McaRPPGFSAFK(Dnp)-OH (for TLN, PLN and ALN) and McaPLGL(Dpa)AR-NH_2_ (for MMP-9 and MMP-14). The concentration of the substrates used was 4 µM except for ALN where it was 5 μM and the highest concentration of the inhibitor compounds tested was 100 µM. *K*_i_ values were only obtained for compounds where 100 µM of the compound reduced the enzymatic activity by 60% or more, as described in the main text. Compound and enzyme were pre-incubated for 30 min at room temperature and the reaction was started by the addition of substrate as described in Materials and Methods.

Compound	*K*_i_ ± s.d. (μM)				
	McaPLGL(Dpa)AR-NH_2_		McaRPPGFSAFK(Dnp)-OH		
	MMP-14	MMP-9	ALN	PLN	TLN
TPA	1.2 ± 0.1	15 ± 4	16 ± 1	5 ± 2	5.5 ± 0.9
Zn146	3.8 ± 0.3	21 ± 5	25 ± 2	4 ± 1	n.d. ^a^
Zn145	1.5 ± 0.1	n.d.	n.d.	12 ± 4	n.d.
Zn230	3.5 ± 0.3	22 ± 6	20 ± 2	1.1 ± 0.3	12 ± 2
Zn148	8.6 ± 0.7	n.d.	n.d.	5 ± 2	n.d.
Zn225	4.5 ± 0.4	28 ± 7	n.d.	5 ± 2	9 ± 2
Zn224	4.5 ± 0.4	n.d.	n.d.	13 ± 4	n.d.
Zn227	4.8 ± 0.4	77 ± 19	n.d.	9 ± 3	11 ± 2
Zn244	4.0 ± 0.3	22 ± 6	n.d.	4 ± 1	n.d.
Zn155	2.4 ± 0.2	26 ± 6	60 ± 6	6 ± 2	n.d.
Zn177	11.2 ± 0.9	56 ± 14	n.d.	11 ± 4	n.d.
Zn175	n.d.	6 ± 2	14 ± 1	7 ± 2	n.d.
DPA	n.d.	n.d.	n.d.	4 ± 1	n.d.
Zn239	6.1 ± 0.5	58 ± 14	n.d.	5 ± 2	32 ± 5
Zn45	2.5 ± 0.2	n.d.	n.d.	2.8 ± 0.9	7 ± 1

^a^ n.d., not determined.

## Data Availability

Not applicable.

## Supplementary Materials

The following are available online. Figure S1: Time-dependent inhibitory effect of 100 μM of TPA derivatives on MMP-9, MMP-14, TLN, PLN and ALN. Figure S2: Time-dependent inhibitory effect of 100 μM of DPA, TPEN, pyridine and thiophene derivatives on the activity of MMP-9, MMP-14, TLN, PLN and ALN.
