# Supplementary material for: Zinc-Chelating Compounds as Inhibitors of Human and Bacterial Zinc Metalloproteases
Source: Molecules. 2021 Dec 22;27(1):56. doi: 10.3390/molecules27010056 (PMC8746695; doi:10.3390/molecules27010056)
Supplement: Supplementary file 1 [file molecules-27-00056-s001.zip › molecules-1501496-supplementary.pdf]

Supplementary Material

Zinc-chelating compounds as inhibitors of human and bacterial zinc metalloproteases

Fatema Rahman[1], Imin Wushur[1], Nabin Malla[1], Ove Alexander Høgmoen Åstrand[2], Pål Rongved[2], Jan-Olof Winberg[1] and Ingebrigt Sylte[1,*]

[1]Molecular Pharmacology and Toxicology, Department of Medical Biology, faculty of Health Sciences, UiT —The Arctic University of Norway, NO-9037 Tromsø, Norway; fatema.rahman@uit.no (FR); imin.wushur@uit.no (IW); nabin.malla@uit.no (NM); ingebrigt.sylte@uit.no (IS); jan.o.winberg@uit.no (JOW).
[2]Department of Pharmaceutical Chemistry, School of Pharmacy, University of Oslo, PO Box 9 1068 Blindern, N-0316 Oslo, Norway; alexander.aastrand@gmail.com (OAHÅ); pal.rongved@farmasi.uio.no (PR).

Correspondence: ingebrigt.sylte@uit.no; Tel.: +47-77644705

Supplementary figure S1

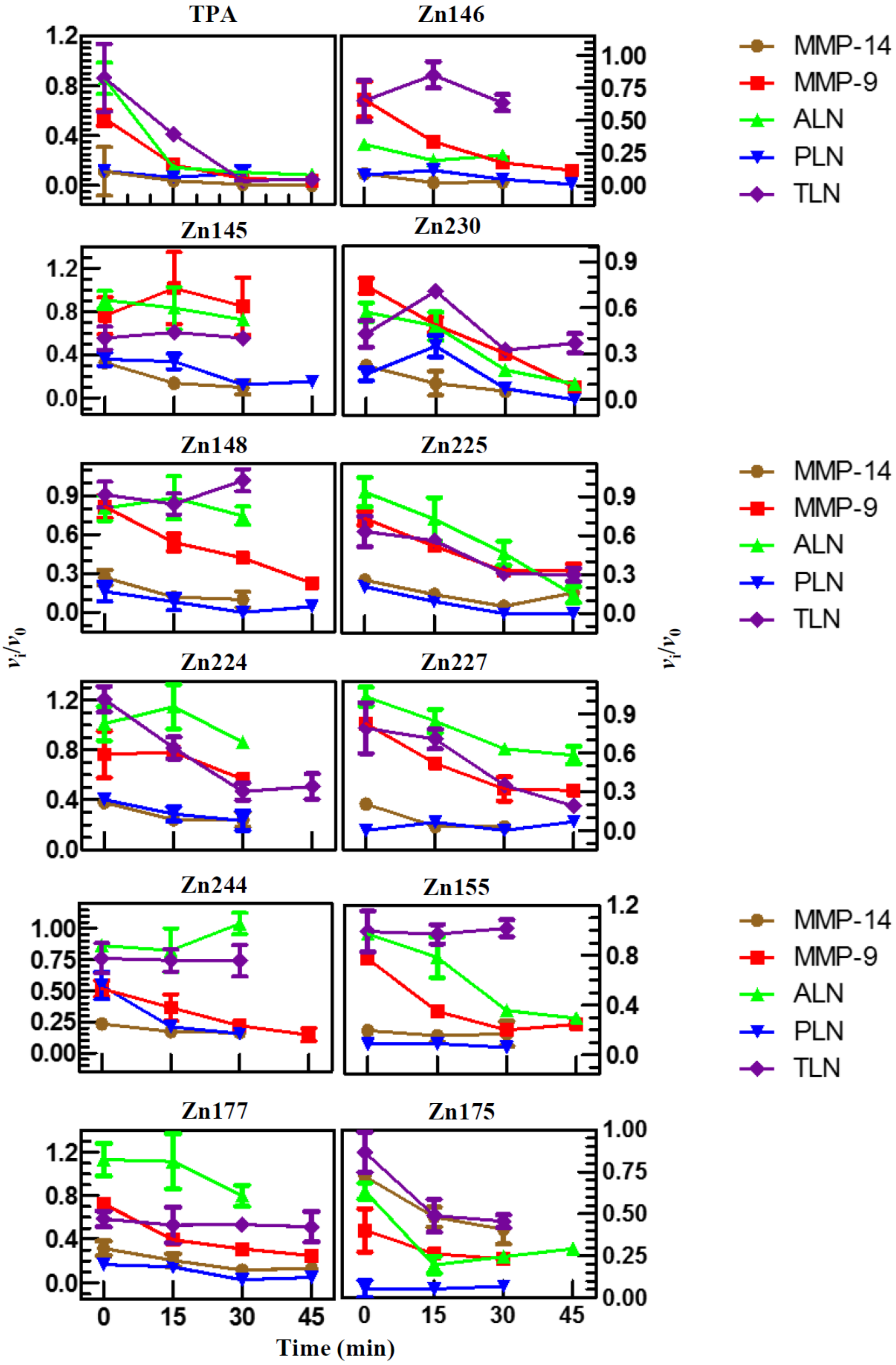

Figure S1. Time dependent inhibitory effects of 100 μM TPA derivatives on the activity of MMP-9, MMP-14, TLN, PLN and ALN. The inhibition experiments were performed as described in Materials and Methods using a concentration of 4 μM of the MMP-9 and MMP-14 substrate McaPLGL(Dpa)AR-NH_2 and the ALN, PLN and TLN substrate McaRPPGFSAFK(Dnp)-OH, except for ALN, where 5 μM was used. The v_i/v_0 (mean ± s.d.) were based on 3-6 experiments.

Supplementary figure S2

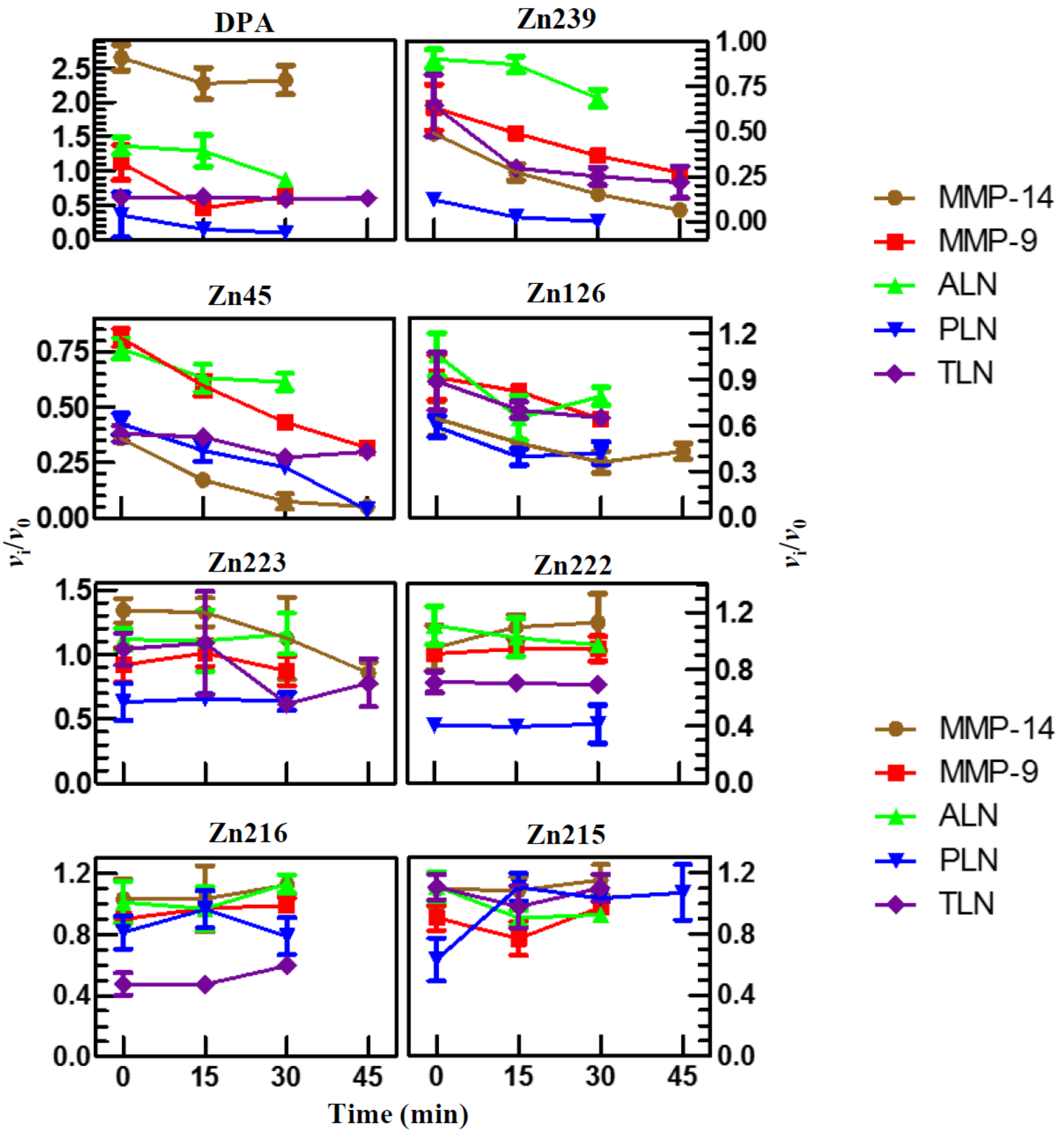

Figure S2. Time dependent inhibitory effects of 100 μM DPA, TPEN, pyridine and thiophene derivatives on the activity of MMP-9, MMP-14, TLN, PLN and ALN. The inhibition experiments were performed as described in Materials and Methods using a fixed concentration of 4 μM of the MMP-9 and MMP-14 substrate McaPLGL(Dpa)AR-NH_2 and the ALN, PLN and TLN substrate McaRPPGFSAFK(Dnp)-OH. For ALN 5 μM was used. The v_i/v_0 (mean ± s.d.) were based on 3-6 experiments.
